# Authors’ Reply: Methodological Considerations in Evaluating Mental Health Apps: Ensuring Reliability and Patient Safety

**DOI:** 10.2196/85910

**Published:** 2025-11-19

**Authors:** Seema Mehrotra, Ravikesh Tripathi, Pramita Sengupta, Abhishek Karishiddimath, Angelina Francis, Pratiksha Sharma, Paulomi Sudhir, T K Srikanth, Girish Rao, Rajesh Sagar

**Affiliations:** 1 Department of Clinical Psychology, National Institute of Mental Health and Neurosciences, Hosur Road, Bengaluru, 560029, India, 91 9448503853; 2 E-Health Research Centre, International Institute of Information Technology Bangalore, Bengaluru, India; 3 Centre for Public Health, Department of Epidemiology, National Institute of Mental Health and Neurosciences, Bengaluru, India; 4 Department of Psychiatry, All India Institute of Medical Sciences, New Delhi, India

**Keywords:** mental health apps, mHealth, review of apps, smartphone apps, MHapps for indian users, India

We thank Balakrishna [1] for the letter to the editor in response to our article [2] on the systematic review of mental health apps accessible to Indian users and appreciate both their interest and the critical points raised.

As described in our paper, we adopted a multiphased approach to interrater reliability. This included initial training of reviewers; independent rating of 3 apps by 4 primary reviewers, followed by joint discussions with mentors; joint review of 3 additional apps, leading to the creation of an internal guide; independent review of 6 more apps and further mentor discussions; detailed independent review of the remaining apps; and mentor review of a subset of 7 reviewed apps, along with joint rating of all apps flagged as doubtful [2]. We acknowledge the concern regarding the absence of intraclass correlation coefficients due to the lack of dual ratings across all apps. It is a limitation noted in our paper. Given the broad search strategy, with use of 15 mental health–related terms, we had a large set of apps to review even after initial screening (n=792). Moreover, our review extended beyond the Mobile Application Rating Scale to include additional predefined parameters, requiring substantial time and effort. The dynamic nature of app store content further constrained the timeline, limiting the feasibility of dual independent ratings for all apps. Nonetheless, we believe our sequential, collaborative approach helped mitigate rating inconsistencies to a reasonable extent. We appreciate the suggestion to report intraclass correlation coefficients for a random subset, and this exercise is already underway.

Regarding sampling scope, as noted in the Limitations section of our paper, we evaluated only free apps, apps offering free trials, and the freely accessible portions of paid apps. This may have led to an incomplete basis for rating certain apps, and we agree that our findings may not fully represent the broader app ecosystem. However, information regarding involvement of mental health professionals and empirical research was drawn from app store descriptions and “about the app” sections, which were accessible even for paid apps. Our focus on free and partially free apps reflects what is most accessible to users and our findings align with patterns observed in other reviews [3-5]. That said, we acknowledge that ratings on some parameters would have benefited from access to full app content. This review is part of a planned recurring exercise, and in the next phase we will conduct detailed assessments of a random sample of fully paid apps, along with newer entries in this dynamic and evolving space.

Lastly, we concur with the observation that adverse events in clinical trials of mental health apps remain underreported. Ours was a review study, and we hope that our identification of certain instances of unscientific or potentially misleading content will prompt further research into these aspects. Looking ahead, we are developing a web-based platform to guide users and professionals on key cautionary indicators—contextually relevant cues to assess app quality and support informed decision-making.
